# Biomechanical comparison of the therapeutic effect of a novel proximal femoral bionic intramedullary nail and traditional inverted triangle hollow screw on femoral neck fracture

**DOI:** 10.1186/s13018-024-04770-4

**Published:** 2024-06-17

**Authors:** Yi-Fan Zhang, Chuan Ren, Meng-Xuan Yao, Shu-Guang Zhao, Kai Ding, Hai-Cheng Wang, Wei Chen, Qi Zhang, Ying-ze Zhang

**Affiliations:** 1 Department of Orthopaedic Surgery, Third Hospital of Hebei Medical University, Shijiazhuang, 050051 Hebei People’s Republic of China; 2 Chinese Academy of Engineering, Beijing, People’s Republic of China

**Keywords:** Femoral Neck fracture, Bionic internal fixation, Triangular support fixation, Biomechanics

## Abstract

**Objective:**

A novel Proximal Femoral Bionic Nail (PFBN) has been developed by a research team for the treatment of femoral neck fractures. This study aims to compare the biomechanical properties of the innovative PFBN with those of the conventional Inverted Triangular Cannulated Screw (ITCS) fixation method through biomechanical testing.

**Methods:**

Sixteen male femoral specimens preserved in formalin were selected, with the donors’ age at death averaging 56.1 ± 6.3 years (range 47–64 years), and a mean age of 51.4 years. The femurs showed no visible damage and were examined by X-rays to exclude diseases affecting bone quality such as tumors, severe osteoporosis, and deformities. The 16 femoral specimens were randomly divided into an experimental group (*n* = 8) and a control group (*n* = 8). All femurs were prepared with Pauwels type III femoral neck fractures, fixed with PFBN in the experimental group and ITCS in the control group. Displacement and stress limits of each specimen were measured through cyclic compression tests and failure experiments, and vertical displacement and strain values under a 600 N vertical load were measured in all specimens through vertical compression tests.

**Results:**

In the vertical compression test, the average displacement at the anterior head region of the femur was 0.362 mm for the PFBN group, significantly less than the 0.480 mm for the ITCS group (*p* < 0.001). At the fracture line area, the average displacement for the PFBN group was also lower than that of the ITCS group (0.196 mm vs. 0.324 mm, *p* < 0.001). The difference in displacement in the shaft area was smaller, but the average displacement for the PFBN group (0.049 mm) was still significantly less than that for the ITCS group (0.062 mm, *p* = 0.016). The situation was similar on the posterior side of the femur. The average displacements in the head area, fracture line area, and shaft area for the PFBN group were 0.300 mm, 0.168 mm, and 0.081 mm, respectively, while those for the ITCS group were 0.558 mm, 0.274 mm, and 0.041 mm, with significant differences in all areas (*p* < 0.001). The average strain in the anterior head area for the PFBN group was 4947 μm/m, significantly less than the 1540 μm/m for the ITCS group (*p* < 0.001). Likewise, in the fracture line and shaft areas, the average strains for the PFBN group were significantly less than those for the ITCS group (*p* < 0.05). In the posterior head area, the average strain for the PFBN group was 4861 μm/m, significantly less than the 1442 μm/m for the ITCS group (*p* < 0.001). The strain conditions in the fracture line and shaft areas also showed the PFBN group was superior to the ITCS group (*p* < 0.001). In cyclic loading experiments, the PFBN fixation showed smaller maximum displacement (1.269 mm vs. 1.808 mm, *p* < 0.001), indicating better stability. In the failure experiments, the maximum failure load that the PFBN-fixated fracture block could withstand was significantly higher than that for the ITCS fixation (1817 N vs. 1116 N, *p* < 0.001).

**Conclusion:**

The PFBN can meet the biomechanical requirements for internal fixation of femoral neck fractures. PFBN is superior in biomechanical stability compared to ITCS, particularly showing less displacement and higher failure resistance in cyclic load and failure experiments. While there are differences in strain performance in different regions between the two fixation methods, overall, PFBN provides superior stability.

## Introduction

As the aging population gradually increases, the incidence of femoral neck fractures is also on the rise. Statistics show that the incidence of femoral neck fractures accounts for approximately 3.1% of all fractures, and it is projected to reach 3 million cases by the year 2050, with the elderly accounting for about 90% of these fractures [1–3]. In China, Chen et al [4] conducted a national retrospective large-data survey on traumatic fractures. This study involved 535,836 individuals and found that, in 2014, the national incidence rate of traumatic fractures in China was 3.21 per thousand people (3.65 for males and 2.75 for females), with the incidence rate of traumatic femoral fractures being 0.35 per thousand people. As the population ages, the incidence rate of femoral fractures increases, with the rate in the population over 65 years of age being 1.11 per thousand for men and 1.39 per thousand for women. Additionally, Lv et al [5] conducted a study on the short-term impact of COVID-19 on the risk of traumatic fractures in Chinese cities. The study collected data from 67,249 patients, with femoral fractures accounting for 13.6% of all types of fractures. Another study indicated that the incidence rate of femoral neck fractures in elderly individuals in long-term care facilities was 8.7 to 9.4 times higher than that in elderly individuals living at home [6]. These data suggest that the incidence of femoral neck fractures increases with age, especially among the elderly and hospitalized patients.

Due to the high rate of postoperative complications associated with conservative treatment, early surgical intervention is commonly recommended for patients under the age of 65 suffering from femoral neck fractures. The treatment for femoral neck fractures often involves early internal fixation surgeries, including inverted triangular cannulated screws (ITCS) and dynamic hip screws (DHS) [7–9]. However, postoperative complications such as avascular necrosis of the femoral head, nonunion, and shortening of the femoral neck remain relatively high with the use of ITCS and DHS, with the mortality rate within one year post-surgery reaching up to 30% [10, 11]. The complications following femoral neck fracture surgery are related to the difficulty of internal fixation in restoring the normal biomechanical conduction characteristics of the proximal femur, specifically the mismatch between the design principle of ITCS and the typical anatomical structure and biomechanical characteristics of the proximal femur. The proximal femur has a typical trabecular system, including primary tension and compression trabeculae that adapt to medially directed compression and lateral tension, with the basal part of the femoral neck, the intermediate area, and the subcapital region bearing 40%, 50%, and 70% of the body’s load, respectively [12, 13]. The multiple parallel fixation screws of ITCS provide longitudinal compression fixation for femoral neck fractures but struggle to conduct both tension and compression in the femoral neck simultaneously, leading to stress concentration and fracture instability, which in turn results in a high risk of complications such as femoral neck shortening, varus collapse, and nonunion. Several scholars have added a medial support plate and increased the number of screws to the ITCS base to enhance the compression resistance of the proximal femur, improving stability [14–16]. However, these improvements neglect the conduction of tensile forces and increase tissue damage, with long-term clinical application still being controversial. Current research suggests that postoperative complications are related to treatment time, fracture type, and quality of reduction, with internal fixation itself being an independent risk factor for complications of femoral neck fractures [17, 18]. Biomechanical factors of internal fixation for femoral neck fractures, including poor stability and stress concentration, are important contributors to nonunion and avascular necrosis of the femoral head [19, 20].

In view of the above situation, Dr. Zhang Yingze proposed the concept of bionic intramedullary nail (PFBN) for the proximal femur to simulate the tension and pressure bone trabecular structure of the proximal femur, and added tension screws and fixation screws to form a double triangle fixation to enhance the conduction of pressure and tension. **Existing studies have indicated that PFBN has better mechanical properties than traditional internal fixation in fixing intertrochanteric fractures** [21, 22]. **However, the biomechanical properties of PFBN fixed femoral neck fracture have not been analyzed**, so this study conducted biomechanical research using human femur specimens, aiming to explore the changes of proximal femoral stress distribution and mechanical conduction after PFBN and ITCS fixed femoral neck fracture, and provide biomechanical evidence for the clinical application of PFBN. This study speculated that compared with ITCS, PFBN fixation of femoral neck fractures can optimize the proximal femoral stress distribution and stress conduction, thereby improving the stability of internal fixation.

## Materials and methods

### Experimental equipment and materials

**Fig. 1:**
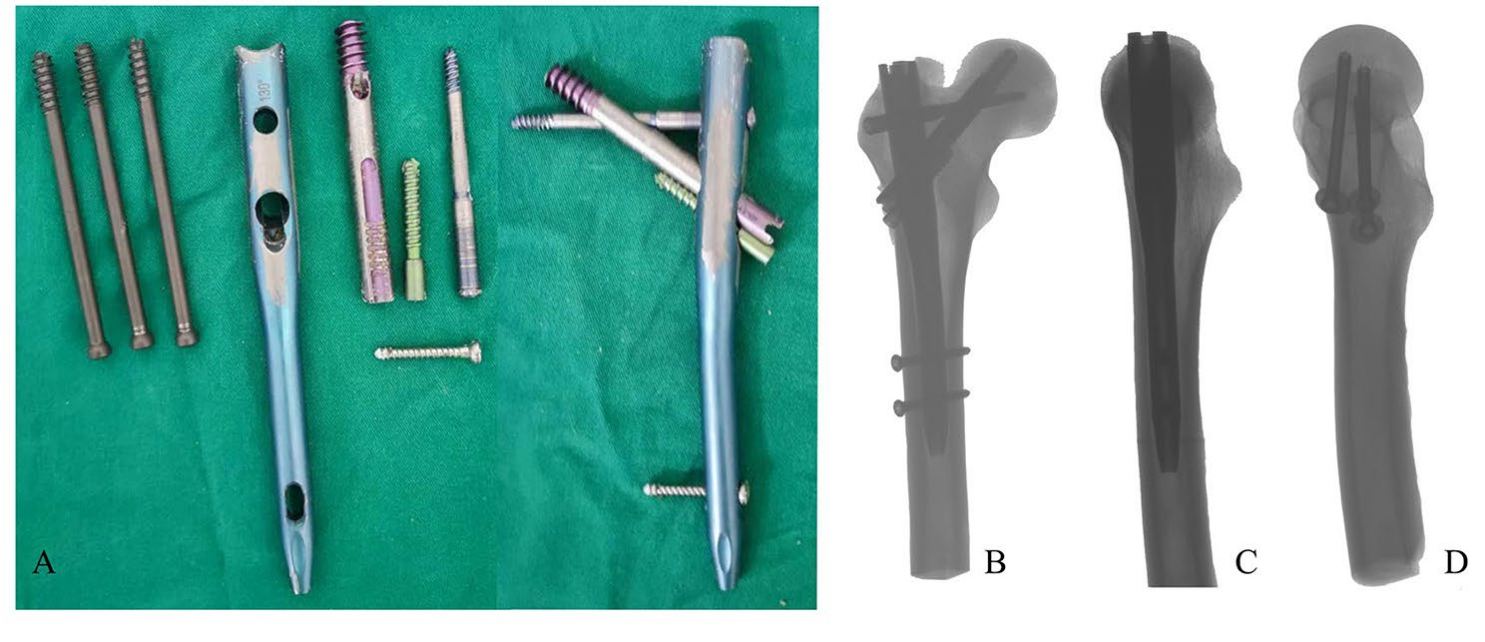
Structure of PFBN and ITCS(**A**) and radiographs of femoral neck fractures fixed by PFBN and ITCS(**B** – **C**)

The study utilized standard surgical instruments and orthopedic tools including several orthopedic traction pins with a diameter of 2 mm, eight sets each of PFBN and ITCS internal fixation systems(Figure 1), and one orthopedic electric drill. Dental impression materials were also used, specifically self-curing liquid resin and self-curing powder resin for dental trays. Biomechanical testing was carried out with a BOSE Electroforce 3520-AT machine (BOSE Corporation, USA) and a global domain strain acquisition system from Gom, Germany.

### Specimen selection

Sixteen male femur specimens preserved in formalin were selected for the study. The donors had an average age of death of 56.1 ± 6.3 years (range of 47–64 years). The specimens were provided by Henan Xuchang Yulin Scientific and Educational Equipment Co., Ltd. Prior to the study, each femur was inspected via X-ray to exclude any bone quality affecting conditions such as tumors, severe osteoporosis, or deformities. Soft tissues, including the skin, muscle, and periosteum, were removed from the femurs, which were then sectioned in the mid-shaft area 25 cm from the femoral head. Post-dissection, the specimens were wrapped in polyethylene film to prevent dehydration and stored at -20 °C. Twelve hours before the experiment, the specimens were thawed at room temperature.

### Experimentation process

#### Creation of femoral neck fracture models and internal fixation implantation

**Fig. 2:**
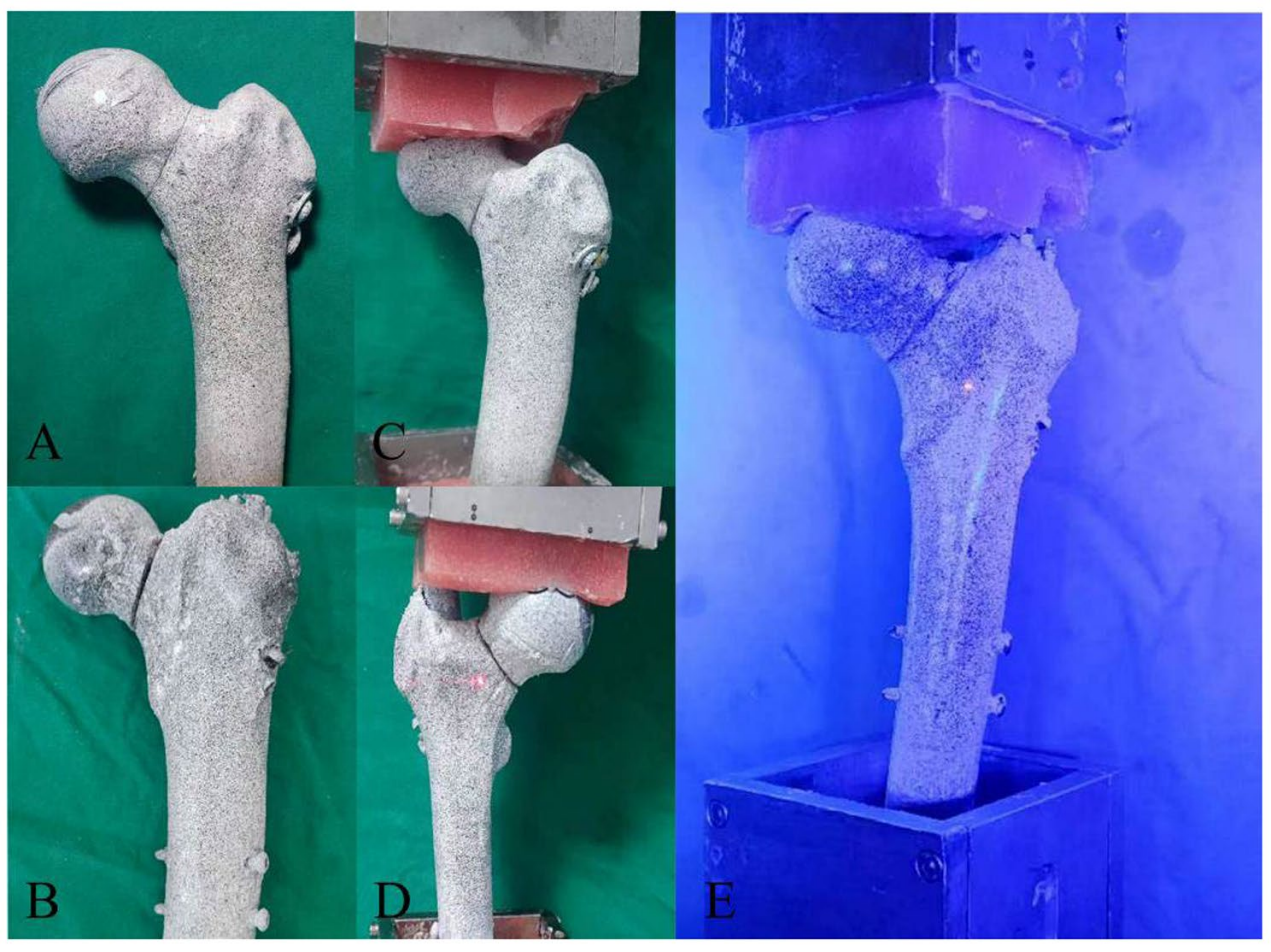
Spray painting pretreatment of femur specimen(**A** – **D**) and experimental photos(**E**)

The sixteen femoral specimens were randomly divided into two groups: an experimental group (*n* = 8) and a control group (*n* = 8). All femurs were modeled to create a Pauwels type III femoral neck fracture. The experimental group was treated with PFBN internal fixation, while the control group received ITCS internal fixation. The success of the modeling was confirmed by gross measurement and X-ray examination (Fig. 1). The surfaces of the experimental specimens were then sanded and cleaned with ethanol and acetone, and allowed to dry naturally. A paint pretreatment was performed on the surface of the normal femoral specimens (Fig. 2) to enable the German Gom global domain strain acquisition system to recognize the marked points on the specimen surfaces.

### Fixation of the experimental specimens

Each femur’s distal end was placed at a 7° incline (simulating the normal anatomical position of the human body) within a fixture, ensuring it was centered within the fixture’s curved groove. The self-curing dental tray liquid and powder were mixed to a paste-like consistency and poured into the fixture until the femur was completely embedded, maintaining the relative position of the femur for about 15 min. Once the femur was firmly fixed, the fixture was attached to the BOSE Electroforce 3520-AT biomechanical testing machine (Fig. 2). The self-curing dental tray liquid and powder were then mixed evenly and poured into a mold. As the mixture began to set, it was fixed in the biomechanical testing machine and pressed against the femoral head to simulate the creation of an acetabular model, keeping the two molds in a fixed relative position for 15 min.

### Experimental procedure

#### Cyclic loading test

Specimens were fixed on the biomechanical testing machine at a 7° incline angle using the fixture (Fig. 2), and subjected to 10,000 cycles at a frequency of 2 Hz. The valley load for each cycle was maintained at 200 N, and the peak load was maintained at 600 N, in order to simulate the weight-bearing conditions after internal fixation of a femoral neck fracture.

#### Vertical compression test

The specimens were fixed in the biomechanical testing machine, and a preload of 200 N was applied vertically for 2 min before testing to eliminate elastic creep. The vertical load was then increased from 0 N at a rate of 5 N/s up to 600 N. Once the peak load was reached, it was maintained for 2 min. Throughout this process, the strain on the specimen was measured and recorded using an optical three-dimensional deformation tracking measurement system.

#### Failure experiment

The femur was compressed vertically at a speed of 2 mm/min, with real-time recording of axial load and displacement data. The specimen was considered to have failed when internal fixation loosening, fracture, screw cut-out, a new fracture, or a sudden drop in axial load occurred.

### Data collection

**Fig. 3:**
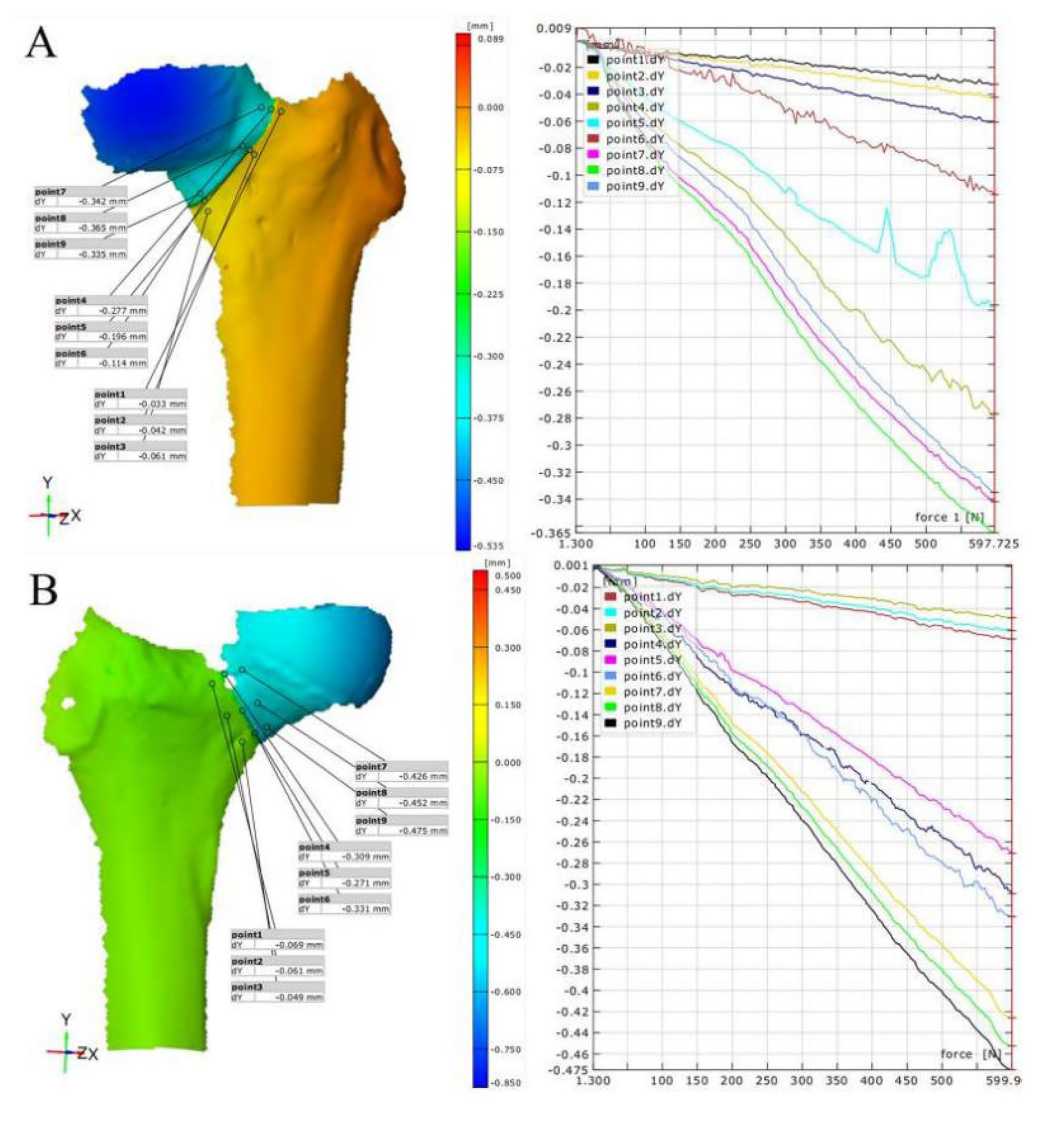
Displacement Contour and Line Graphs for PFBN (**A**) and ITCS (**B**) on the Anterior Aspect

During the cyclic loading test, the maximum inter-fragmentary displacement during the loading process was recorded. If the specimen’s internal fixation failed before completing 10,000 cycles, the number of cycles completed was recorded. In the vertical compression test, the overall and local strain of the samples was measured using the Gom global domain strain acquisition system. Points 1 to 9 were established along the direction of the fracture line, at the center and on both sides of the fracture line (Fig. 3). The vertical displacement and strain values of these 9 points at 600 N stress were recorded. Points 1 to 3 represent the average vertical displacement and strain of the femoral shaft side, also known as the shaft area, at the fracture line. Points 4 to 6 reflect data around the fracture line, the fracture zone, and points 7 to 9 represent the displacement and strain values on the femoral head side, also known as the head area, of the fracture line. The failure experiment recorded the maximum failure load for all 16 femurs from both groups.

**Fig. 4:**
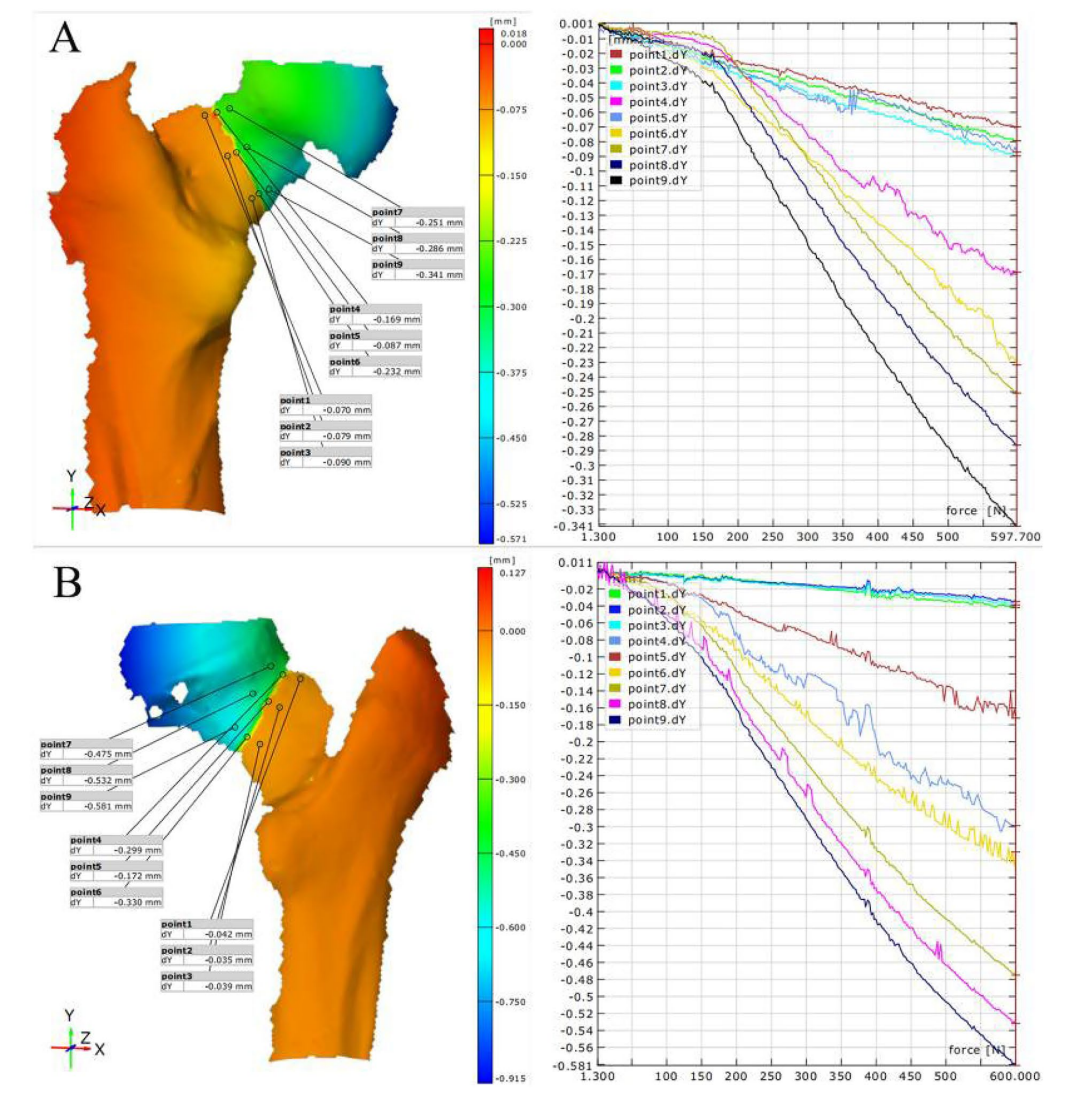
Displacement Contour and Line Graphs for PFBN (**A**) and ITCS (**B**) on the Posterior Aspect

### Data analysis

The GraphPad Prism 8 statistical software was used to organize and analyze the experimental data. The data from this experiment were quantitative and were tested for normality and homogeneity of variance using the Shapiro-Wilk and Levene methods, respectively. An unpaired t-test was used to detect differences in mechanical parameters between the experimental and control groups. A P-value of < 0.05 was considered statistically significant.

## Result

### Vertical compression test: PFBN vs. ITCS displacement situations

In this study, we compared the vertical displacement of different areas on the anterior and posterior sides of the femur under a 600 N force for two types of femoral neck fracture internal fixation methods: PFBN and ITCS(Figs. 3 – 4). The specific test regions included the fracture line zone, the head region near the femoral head, and the shaft region near the femoral shaft. The research findings are as follows: In the anterior head region, the average displacement of the PFBN group was 0.362 ± 0.071 mm, compared to the ITCS group, which had an average displacement of 0.480 ± 0.084 mm. Statistical analysis showed a significant difference between the two groups (t = 5.335, *p* < 0.001) (Table 1).In the fracture line zone, the average displacement of the PFBN group was 0.196 ± 0.088 mm, while the ITCS group had an average displacement of 0.324 ± 0.068 mm. The displacement difference between the groups in this region was also significant (t = 5.592, *p* < 0.001) (Table 1).In the shaft region, the average displacement for the PFBN group was 0.049 ± 0.020 mm, and for the ITCS group, it was 0.062 ± 0.017 mm. Although the displacement difference here was smaller, it was still statistically significant (t = 2.499, *p* = 0.016) (Table 1).On the posterior head region, the PFBN group had an average displacement of 0.300 ± 0.093 mm, while the ITCS group had an average displacement of 0.558 ± 0.105 mm. There was a significant difference between the groups (t = 8.991, *p* < 0.001) (Table 2).In the fracture line zone on the posterior side, the PFBN group had an average displacement of 0.168 ± 0.081 mm, and the ITCS group had an average displacement of 0.274 ± 0.010 mm. The difference in displacement for this region was statistically significant (t = 4.080, *p* < 0.001) (Table 2).In the shaft region on the posterior side, the average displacement for the PFBN group was 0.081 ± 0.027 mm, compared to the ITCS group, which had an average displacement of 0.041 ± 0.014 mm. This difference was also significant (t = 6.503, *p* < 0.001) (Table 2).

**Table 1:** Comparison of displacement in different anterior areas between the two groups (mm)

	PFBN	ITCS	t	*p*
head area	0.362 ± 0.071	0.480 ± 0.084	5.335	<0.001
fracture area	0.196 ± 0.088	0.324 ± 0.068	5.592	<0.001
shaft area	0.049 ± 0.020	0.062 ± 0.017	2.499	0.016

**Table 2:** Comparison of displacement in different posterior areas between the two groups (mm)

	PFBN	ITCS	t	*p*
head area	0.300 ± 0.093	0.558 ± 0.105	8.991	<0.001
fracture area	0.168 ± 0.081	0.274 ± 0.010	4.08	<0.001
shaft area	0.081 ± 0.027	0.041 ± 0.014	6.503	<0.001

### Vertical compression test: PFBN vs. ITCS strain situations

**Fig. 5:**
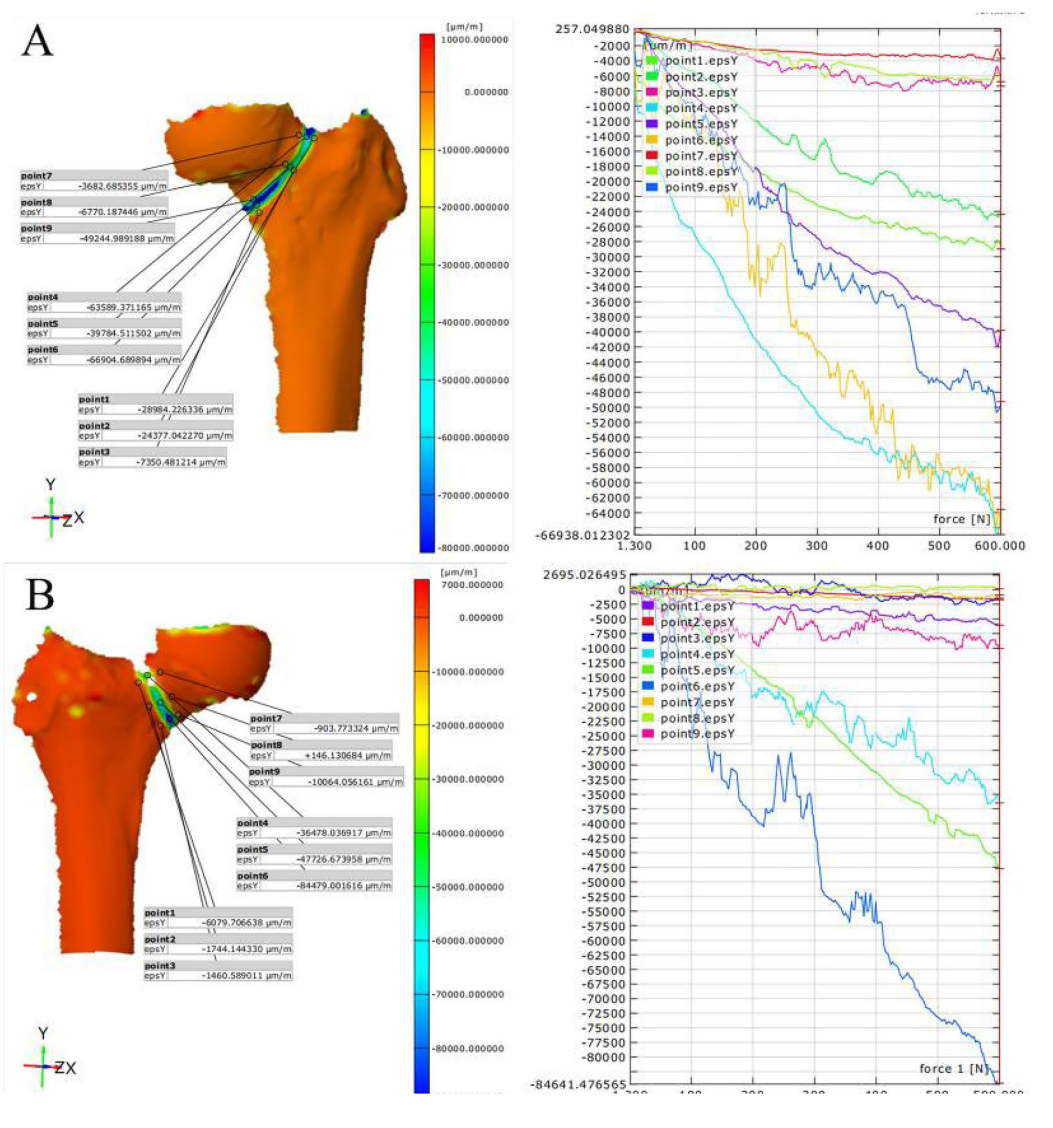
Strain Contour and Line Graphs for PFBN (**A**) and ITCS (**B**) on the Anterior Aspect

**Fig. 6:**
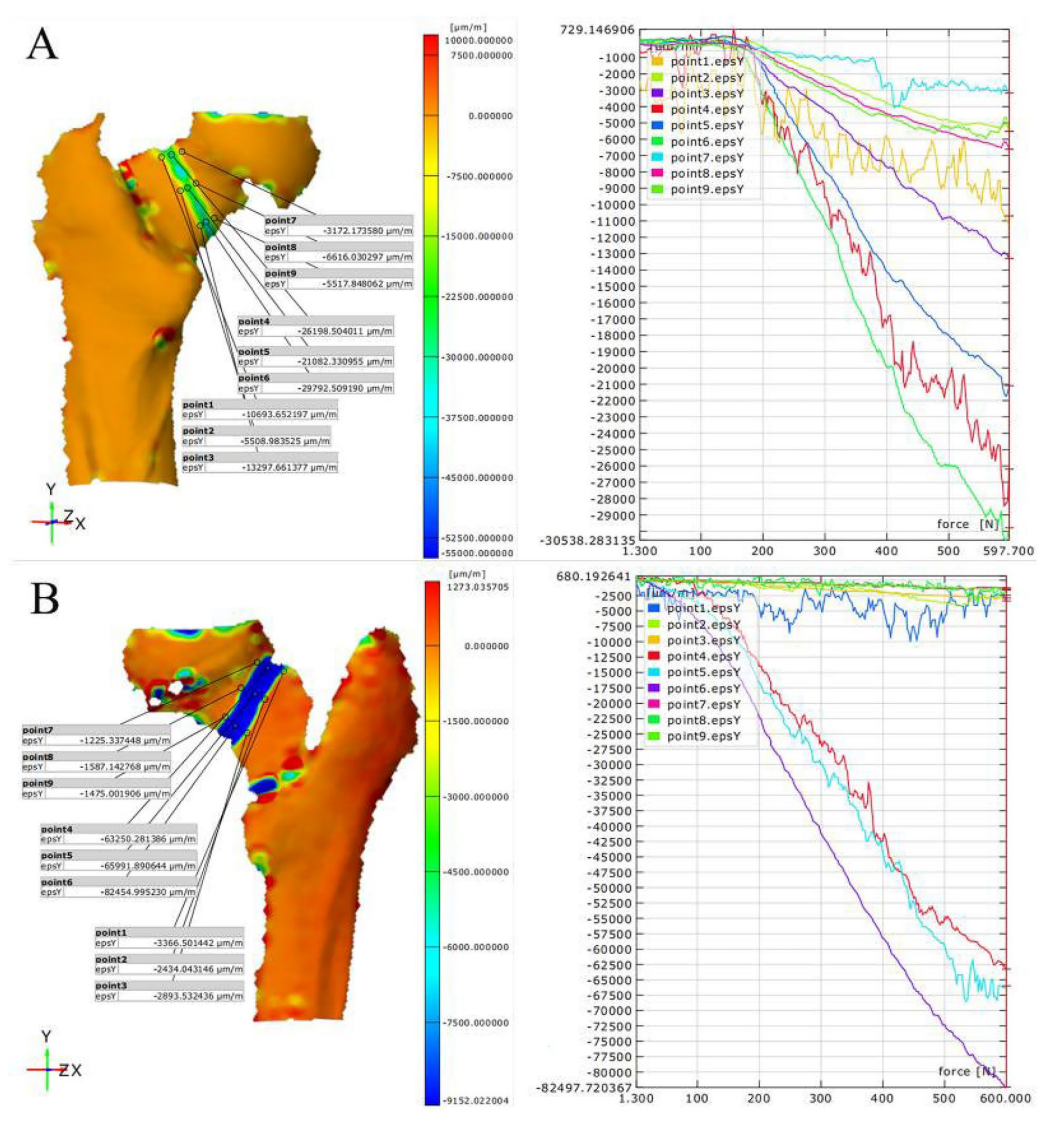
Strain Contour and Line Graphs for PFBN (**A**) and ITCS (**B**) on the Posterior Aspect

In this biomechanical study, the strain in different regions on the anterior and posterior sides of the femur under a 600 N force was measured for two types of femoral neck fracture internal fixation methods: PFBN and ITCS (Fig. 5 – 6). The results are as follows: In the anterior head region, the average strain for the PFBN group was 4947 ± 2833 μm/m, while for the ITCS group, the average strain was 1540 ± 1394 μm/m, with a statistically significant difference between the groups (t = 4.317, *p* < 0.001) (Table 3).In the fracture line zone, the average strain for the PFBN group was 50,678 ± 14,085 μm/m, compared to the ITCS group, which had an average strain of 65,984 ± 25,790 μm/m. The difference in strain between the two was significant (t = 2.552, *p* = 0.014) (Table 3).In the shaft region, the average strain for the PFBN group was 20,404 ± 12,610 μm/m, whereas for the ITCS group, the average strain was 3185 ± 3051 μm/m. The difference in strain between the two was very significant (t = 6.502, *p* < 0.001) (Table 3).On the posterior head region, the average strain for the PFBN group was 4861 ± 2282 μm/m, and for the ITCS group, it was 1442 ± 608.6 μm/m. There was a significant difference between the groups (t = 7.092, *p* < 0.001) (Table 4).In the fracture line zone on the posterior side, the average strain for the PFBN group was 24,252 ± 10,262 μm/m, while for the ITCS group, it was 65,694 ± 29,869 μm/m. The difference in strain for this region was also significant (t = 6.428, *p* < 0.001) (Table 4).In the shaft region on the posterior side, the average strain for the PFBN group was 9544 ± 5077 μm/m, compared to the ITCS group, which had an average strain of 2932 ± 1258 μm/m. The difference between the two groups in the shaft region was also significant (t = 6.193, *p* < 0.001) (Table 4).

**Table 3:** Comparison of strain in different anterior areas between the two groups (µm/m)

	PFBN	ITCS	t	*p*
head area	4947 ± 2833	1540 ± 1394	4.317	<0.001
fracture area	50,678 ± 14,085	65,984 ± 25,790	2.552	0.014
shaft area	20,404 ± 12,610	3185 ± 3051	6.502	<0.001

**Table 4:** Comparison of strain in different posterior areas between the two groups (µm/m)

	PFBN	ITCS	t	*p*
head area	4861 ± 2282	1442 ± 608.6	7.092	<0.001
fracture area	24,252 ± 10,262	65,694 ± 29,869	6.428	<0.001
shaft area	9544 ± 5077	2932 ± 1258	6.193	<0.001

### Cyclic loading and failure experiments

In the biomechanical comparative study of femoral neck fractures using two internal fixation methods, PFBN and ITCS, we evaluated the fracture stability after fixation with cyclic loading and failure experiments. The study results showed significant differences in the performance of the two fixation methods under repeated stress and ultimate failure loads.During cyclic loading tests, fractures fixed with the PFBN method showed smaller maximum displacement values, with an average displacement of 1.269 ± 0.064 mm. In contrast, fractures fixed with the ITCS method had an average displacement of 1.808 ± 0.102 mm (Table 5). Statistical analysis results (t = 12.68, *p* < 0.001) indicated that this difference is highly significant, meaning that under the same cyclic loading conditions, fractures fixed with PFBN were more stable than those fixed with ITCS, displaying a greater ability to withstand repeated loads.Furthermore, in the failure experiments, we assessed the performance of the two internal fixation methods under ultimate loads. The results showed that the maximum failure load that fractures fixed with PFBN could withstand was significantly higher than that for ITCS, with values of 1817 ± 49.83 N and 1116 ± 41.19 N (Table 5), respectively. The difference was significant (t = 30.67, *p* < 0.001).

**Table 5:** Comparison of the maximum displacement values of the fracture blocks under cyclic loading and the maximum failure load in the destruction test between the two groups

	PFBN	ITCS	t	*p*
Cyclic Loading Test(mm)	1.269 ± 0.064	1.808 ± 0.102	12.68	<0.001
Failure Experiment(N)	1817 ± 49.83	1116 ± 41.19	30.67	<0.001

## Discussion

The study has yielded definitive conclusions by comparing the biomechanical stability of two internal fixation methods for femoral neck fractures: the Proximal Femoral Nail (PFBN) and Intramedullary Trochanteric Screw (ITCS). During cyclic loading experiments, the displacement of fracture fragments fixed by PFBN was significantly less than that of ITCS, indicating that PFBN provides better stability under repeated stress. Additionally, in the failure experiments, PFBN withstood a maximum load that was substantially higher than ITCS, further proving its higher resistance to failure under extreme load conditions.In the vertical compression tests, the vertical displacement in the anterior region for ITCS fixation was greater in both the head and fracture line regions compared to PFBN, suggesting that PFBN may offer better stability in these areas. In the posterior region, ITCS also exhibited greater vertical displacement in the head region compared to PFBN, but PFBN showed greater displacement in the shaft region than ITCS. This could indicate that the fixation effect of PFBN in the shaft area is not as good as that of ITCS.In the anterior and posterior regions of the femur, the strain for the PFBN fixation method in the head and shaft areas was significantly higher than in the ITCS group. Conversely, in the fracture line area, the strain was significantly higher for ITCS than for PFBN. This indicates that the PFBN fixation method sustains greater deformation in the head and shaft areas, while the ITCS fixation method experiences greater strain in the fracture line area.These results are clinically relevant when choosing the appropriate internal fixation method for femoral neck fractures, particularly considering the stability of the fixation and the potential stress distribution the fixated area may endure.

Research has shown that PFBN has significant advantages over traditional internal fixation methods in restoring fractures of the proximal femur. Sun et al [23] compared the outcomes of 56 intertrochanteric femoral fractures treated with PFBN and hip replacement surgery (HR), and found no difference between the two groups in terms of surgery time, preoperative and postoperative hemoglobin levels, and postoperative Harris scores at 3 months. Compared to the HR group, the PFBN group had lower total costs, shorter hospital stays, and lower mortality rates. Chen et al [24]compared the biomechanical effectiveness of PFBN and Dynamic Hip Screw (DHS) in treating intertrochanteric femoral fractures, and the results showed that intramedullary fixation is more stable than extramedullary fixation, and PFBN offers better biomechanical stability compared to DHS.ITCS is commonly used to treat femoral neck fractures, but the rate of postoperative complications remains high, with a one-year postoperative mortality rate reaching up to 30%. In this study, the displacement of fracture fragments under cyclic loading in the ITCS group was 1.4 times that of the PFBN group, and in the failure experiments, the maximum stress sustained by PFBN was 1.6 times that of ITCS. These results indicate that PFBN has higher resistance to destruction and can provide greater stability under repeated stress in everyday life compared to ITCS. The study suggests that PFBN, with its stronger mechanical performance and stability, may reduce the incidence of postoperative complications such as avascular necrosis of the femoral head, nonunion, and shortening of the femoral neck in femoral neck fracture surgery.

Current research on the internal fixation treatment of femoral neck fractures is focused on enhancing the biomechanical performance of ITCS by improving various aspects such as the diameter and number of screws. Regarding screw diameter, the study by Lou Yuliang et al [25] found that using ITCS with diameters of 6.5 mm and 8.0 mm to fix femoral neck fractures did not show a statistically significant difference in the rate of fracture healing and postoperative complications such as avascular necrosis of the femoral head. Concerning the number of screws, He Xiaojun et al [26] used 15 frozen human femoral samples to create femoral neck fracture models with different Pauwels angles and fixed them with either two or three ITCS to assess biomechanical stability. The results indicated that using only two ITCS screws for Pauwels type III fractures was insufficient in terms of stability and resistance to torsion. However, more screws are not always better, as the study pointed out that adding a fourth ITCS did not show a biomechanical advantage, which could be due to the increased screw holes reducing the mechanical strength of the lateral femoral wall [27]. At the same time, some scholars have noted that increasing the number of ITCS could damage the internal blood supply of the femoral head, leading to more postoperative complications [28]. The above studies overlooked the insufficient shear resistance of ITCS itself. For unstable femoral neck fractures, such as Pauwels type III, the high vertical shear force can easily lead to postoperative complications such as loss of the neck-shaft angle, shortening of the femoral neck, and internal fixation failure, causing coxa vara deformity and ultimately non-union of the fracture. Simply increasing the diameter and number of screws cannot improve this aspect.

In comparison to the ITCS system, which relies on the theory of converting torsional forces into compressive forces along the axis of the screw to promote contact at the fracture site, the PFBN (Proximal Femoral Bionic Nail) is based on the principle of central fixation, reducing the moment arm and consequently decreasing stress concentration on the bone. The core principle of PFBN involves adding an additional parallel support screw to the existing internal fixation device, the proximal femoral nail antirotation (PFNA). This support screw passes through a hole to form a stable triangular structure (a “metal triangle”) at the proximal end with the tension screw and the main nail. Furthermore, the support and tension screws, combined with the cortical bone at the femoral head and the adjacent cancellous bone, constitute a mixed triangle.Compared to ITCS, the PFBN, with its crossed-structure design, reduces contact stress at the fracture site and decreases stress concentration on the bone. The dual interlocking screw structure prevents the backing out of the head and neck screws and minimizes shortening of the femoral neck, which can be caused by a single screw or parallel screws.Ding et al [29] in a finite element analysis indicated that, compared to ITCS and Dynamic Hip Screws (DHS), PFBN significantly improves in reducing stress concentration, enhancing the distribution of stress, and increasing the overall stability of the femoral neck fracture fixation model. Cheng et al [30] also conducted a finite element analysis on PFBN for fixing femoral neck fractures, and the results showed that the stress within the PFBN fixation was the lowest at 112.0 MPa, significantly lower than the PFNA group (124.8 MPa) and DHS group (149.8 MPa). These two sets of finite element studies are consistent with the results of this biomechanical study: in vertical compression tests, the strain in the fracture line area of the PFBN group was significantly less than that of the ITCS group. In contrast, the strain in the head and shaft areas of the PFBN group was higher than that of the ITCS group, further illustrating that PFBN, by reasonably distributing the stress transmission in the femoral head, increases the contact area, reduces local pressure, avoids stress concentration at the tip of the screw, and minimizes complications such as femoral head cutting and varus collapse of the hip.

This study has certain limitations. Firstly, the study uses cadaveric femur specimens, and there is a gap compared to in vivo experiments. Cadaveric biomechanical experiments cannot guarantee that the fracture line is filled with fibrocartilage tissue similar to what would be found in the human body after a deformity heals, resulting in some deviations from actual conditions. Secondly, the fracture lines in the fracture models created for this study are not completely consistent, which may affect the experimental results. Lastly, variations in the quality of paint applied to different experimental specimens can lead to changes in the sensitivity of the GOM global strain collector in identifying strain points, resulting in some deviations in the experimental data.

## Conclusion

In summary, this study demonstrates that the PFBN offers superior biomechanical stability compared to the ITCS, particularly in cyclic loading and destructive testing, where it showed less displacement and greater resistance to failure. While the two internal fixation methods exhibit differences in strain performance in various regions, overall, the PFBN provides better stability. The PFBN meets the requirements for internal fixation of femoral neck fractures, offering a superior option for clinical selection of internal fixation methods for these types of fractures.

## Data Availability

No datasets were generated or analysed during the current study.
